# Taphonomic patterns mimic biologic structures: diagenetic Liesegang rings in Mesozoic coleoids and coprolites

**DOI:** 10.7717/peerj.10703

**Published:** 2021-01-14

**Authors:** Christian Klug, Gianpaolo Di Silvestro, Rene Hoffmann, Guenter Schweigert, Dirk Fuchs, Thomas Clements, Pierre Gueriau

**Affiliations:** 1 Paläontologisches Institut und Museum, Universität Zürich, Zürich, Switzerland; 2 Trilobite Design Italia, Aurisina (TS), Italy; 3 Institute of Geology, Mineralogy & Geophysics, Ruhr-Universität Bochum, Bochum, Germany; 4 Staatliches Museum für Naturkunde, Stuttgart, Germany; 5 Bayerische Staatssammlung für Paläontologie und Geologie, München, Germany; 6 School of Geography, Earth and Environmental Sciences, University of Birmingham, Birmingham, UK; 7 Institute of Earth Sciences (ISTE), University of Lausanne, Lausanne, Switzerland

**Keywords:** Coleoidea, Vampyropoda, Jurassic, Cretaceous, Diagenesis, Exceptional preservation, Conservation deposits

## Abstract

Because of physiology of coleoids, their fossils preserve soft-tissue-remains more often than other cephalopods. Sometimes, the phosphatized soft-tissues, particularly parts of the muscular mantle, display dark circular patterns. Here, we showcase that these patterns, here documented for fossil coleoids from the Jurassic of Germany and the Cretaceous of Lebanon, superficially resemble chromatophores (which enable living coleoids to alter their coloration). We examined and chemically analyzed the circular structures in these specimens, describe them, and discuss their genesis. Based on their structure and color, we visually differentiate between three types of circles. By comparison with similar structures, we suggest that these structures are not biogenic but Liesegang rings, which formed due to reaction-diffusion processes very soon after death.

## Introduction

Coleoids (octopus, squid and their ancestors) have a surprising record of soft-tissue fossils despite their limited biomineralised tissues. There are known biases that prevent some coleoid tissues from fossilizing (Clements et al., 2016), however, conservation deposits (Konservatlagerstätten sensu Seilacher, 1970) of Carboniferous to Cretaceous age (Naef, 1923; Klinghardt, 1932; Johnson & Richardson, 1968; Rieber, 1970; Kluessendorf & Doyle, 2000; Doguzhaeva, Mapes & Mutvei, 2002; Fuchs, 2006a, 2006b; Schweigert & Dietl, 2010; Donovan, Fuchs & Part, 2016; Klug et al., 2019; Jenny et al., 2019) yield many specimens with phosphatized remains. For example, there are good records of fossilized organs such as the buccal mass, digestive tract, gills, fins, spermatophores, mantle muscles, cephalic cartilage, arm nerve bundles etc. (Keupp et al., 2010; Klug et al., 2015, 2016, 2019; Jenny et al., 2019). The scarcity of soft-tissue preservation in crown group decabrachians is linked with the higher amount of ammonia in their bodies (Clements et al., 2016). Despite this, the anatomy of fossil coleoids is better known than that of ectocochleate cephalopods such as ammonoids (with external shell and presumably low amounts of or no ammonia; Klug, Riegraf & Lehmann, 2012).

Recently, three coleoid specimens and one coprolite were discovered, displaying concentric circular structures. They were found in platy limestones (Plattenkalks) of the German Late Jurassic and the Lebanese Late Cretaceous. Both occurrences are world-renowned conservation deposits, which contain, among many other fossils, reasonably abundant coleoid remains often retaining some remnants of soft-tissue (Allison, 1988; Wilby et al., 2004; Fuchs, 2006a, 2006b; Fuchs & Larson, 2011; Donovan, Fuchs & Part, 2016; Klug et al., 2015, 2016). These soft-tissue remains are usually phosphatized except for jaws and ink, which may be carbonized (Klug et al., 2015).

The specimens under consideration here display more or less circular structures with concentric rings or spirals inside. At first sight, we thought of a first case of fossilized chromatophores, that is, the cells enabling many modern coleoids to rapidly change their colors (Sereni, 1930; Cloney & Florey, 1968; Florey, 1969; Cloney & Brocco, 1983; Dubas et al., 1986; Hanlon, 2007; Kingston et al., 2015; Ramirez & Oakley, 2015). This interpretation was falsified based on the broad size-range of the structures between less than a millimeter and over one centimeter and the absence of characteristic anatomical features, which should be preserved but are not. Additionally, the internal patterns are concentric or spiral, which does not coincide with those of cephalopod chromatophores.

From this falsification, the question for the origin of these patterns arose. Are they comparable to silicification rings (Kazanci & Varol, 1993), Liesegang rings (Keller & Rubinow, 1981; Zhang et al., 2014) or other peculiar mineralization processes (Chernov, 1961; Martins & Rocha, 2007; Cartwright et al., 2009; Seiss, Ouissen & Chaussende, 2013; Shtukenberg et al., 2013)? The structures found in the phosphatized mantle of the Mesozoic coleoids share patterns with all three phenomena.

The aims of this study are to (1) describe all available specimens displaying these phosphatized circular and spiral patterns with a focus on these structures, (2) to document their chemistry and organization, and (3) to discuss their genesis, that is, whether these are biological structures or not and how they formed.

## Material

The specimens are kept in the Paläontologisches Institut und Museum of the Universität Zürich, Switzerland (PIMUZ numbers), as well as at the Staatliches Museum für Naturkunde in Stuttgart, Germany (SMNS numbers). Three coleoids and one coprolite were included here.

The oldest specimens (PIMUZ 51761; SMNS 70496) are from the Beckeri Zone, Ulmense Subzone, *rebouletianum* Horizon (Kimmeridgian) of the Rygol quarry near Painten (Bavaria, Germany). Both belong to *Trachyteuthis*, possibly *T. hastiformis* (Rüppell, 1829), which is one of the more common coleoids of the Solnhofen Archipelago. This assignment is based on the shape of the gladius with a characteristic granulated median field (Fuchs, 2006a, 2006b). The locality has become famous for its exceptionally preserved vertebrates and also for its organic preservation in coleoids (Klug et al., 2015). While chitinous cephalopod mandibles are not preserved in the Solnhofen region, these are commonly present in Painten (Klug et al., 2015).

Like Painten, the Plattenkalk-locality Nusplingen in Baden-Württemberg (Germany) also preserves carbonaceous matter well (slightly older than the fossiliferous strata in Painten). From there, we include the coprolite *Medusites* (Schweigert, 2001), which also shows these circular structures (SMNS P1856).

The last specimen is a complete specimen of *Dorateuthis syriaca* (Woodward, 1883) from the Cenomanian of the Haqel conservation deposit in Lebanon (Fuchs, 2006b; Fuchs & Larson, 2011; Jattiot et al., 2014). This species was identified based on its characteristic elongate gladius and its body proportions. It is also the most common coleoid in this locality. These platy limestones are famous for their articulated fish remains but also for soft-tissue preservation in ammonoids (Wippich & Lehmann, 2004) as well as stomach contents of both ammonoids and nautiloids (Keupp, Saad & Schweigert, 2016).

## Methods

SEM imaging and EDX analyses of thee uncoated samples were done with a Jeol 6010 VP scanning electron microscope primarily at low vacuum conditions and 10 keV. Equipped with a Bruker EDX system we mapped the elements: C, O, Ca, Si, P, F and S with Esprit 2.1 software. In addition, we used Raman spectroscopy with three different laser excitation systems (red, blue, green); however, fluorescence was always too high to obtain useful analytical results.

X-ray diffraction (XRD) was collected on unpreprared millimetric samples extracted from the fossils using a conventional Cu-Kα (λ = 1.5418 Å) diffractometer (ARL X’TRA; Thermo Fisher, Waltham, MA, USA) at the Institute of Earth Sciences, University of Lausanne, using the following experimental conditions: 2θ range: 5–65°; 0.02° angular step, 1 s counting time. Phase identification was carried out using references from the RRUFF database (https://rruff.info/).

Raman microspectroscopy was performed at the Institute of Earth Sciences, University of Lausanne, using a Horiba Jobin Yvon LabRAM 800 HR spectrometer in a confocal configuration, equipped with an Ar+ laser (532 nm) excitation source and an electron multiplying charge-coupled device (EMCCD). Measurements were performed at constant room temperature, directly on the sample surface, by focusing the laser beam with a 300 μm confocal hole using a long working distance ×50 objective (NA = 0.70). This configuration provided a ≈2 μm spot size for a laser power delivered at the sample surface below 1 mW. Light was dispersed using a 1,800 gr/mm diffraction grating.

Most photos were taken with a normal camera (Nikon D6) under white light. One photo was taken using a simple UV-light and a UV-filter on the camera’s lens.

## Description

### Visual classification of circular structures

**Figure 1:**
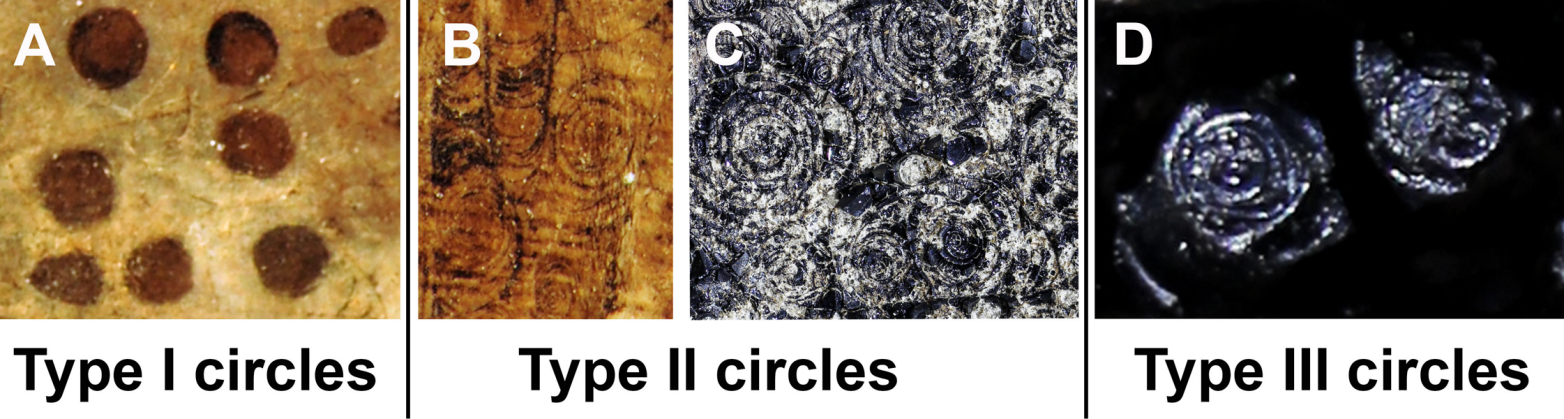
The three types of circles occurring in the fossils described here. The examples are taken from the Figs. 2–4; see further information there. (A) Type I-circles in *Dorateuthis syriaca* (PIMUZ 51762, Cenomanian, Haqel, Lebanon). (B) Type II-circles, same specimen as in A. (C) Type II-circles in *Trachyteuthis hastiformis* (SMNS 70496, Kimmeridgian, Painten, Germany). (D) Type III-circles, same specimen as in C. Photo credit: Dieter Buhl.

This study focuses on circular structures in phosphatic fossils from the Mesozoic. These structures have different appearances. Accordingly, we classified three different forms, here exemplified using specimen SMNS 70496 (types I to III). In the following, we describe these types (overview in Fig. 1):

**Figure 2:**
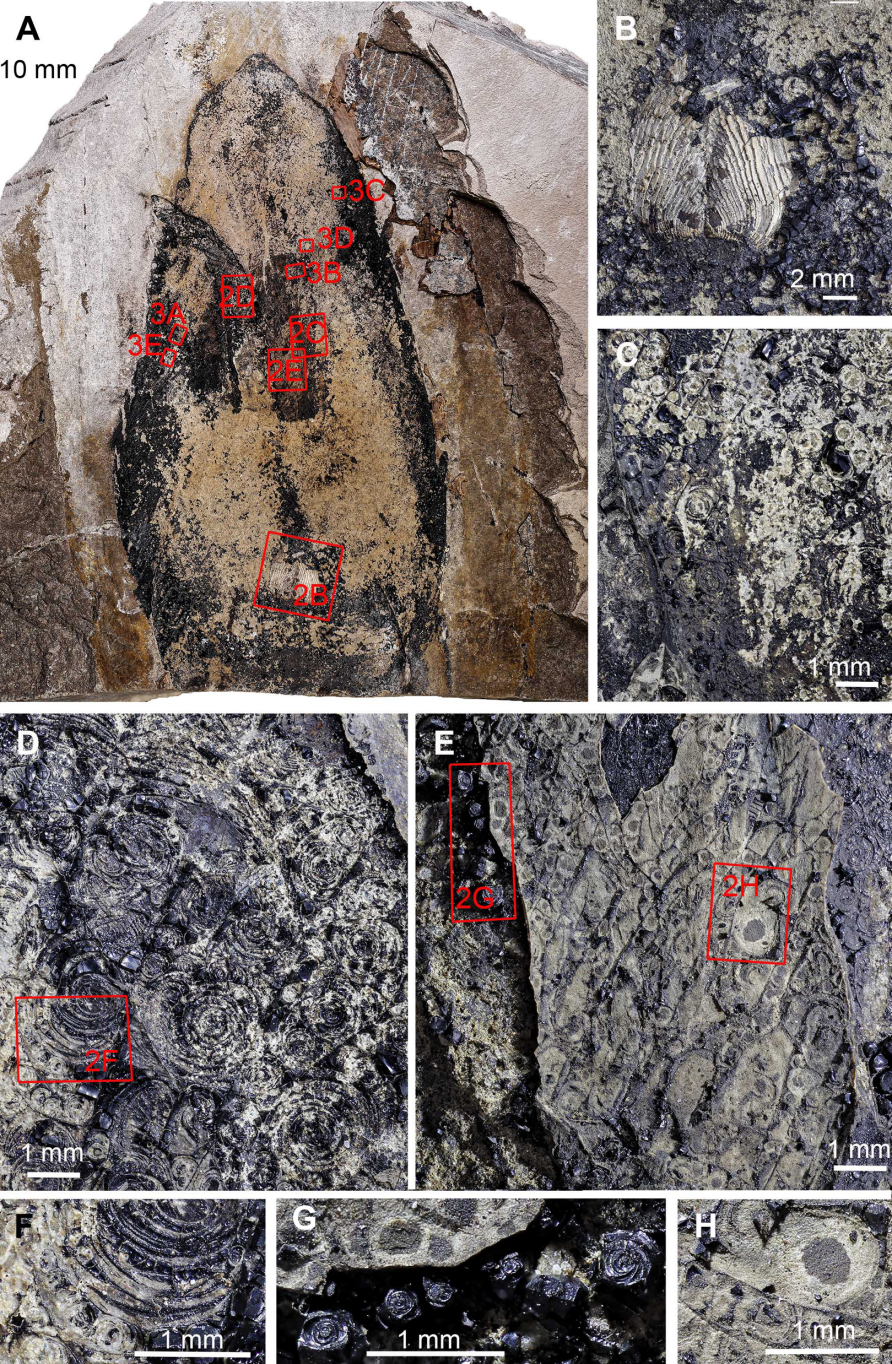
*Trachyteuthis hastiformis*, Kimmeridgian, Painten (Germany). SMNS 70496, leg. M. Kapitzke. The details of A shown in B to H and Fig. 3 are marked with red rectangles. (A) Overview. (B) Detail of *Lamellaptychus* probably in the digestive tract of the animal. (C) Detail showing many light gray Type I-circles with dark gray centers. (D) Concentric Type II-circles rich in organic matter preserved like gagate. (E) Light Type I-circles like in C; note the linear arrangement. (F) Detail of (D) showing Type I- and II-circles of varying sizes (the largest is Type II). (G) Organic rich Type III-circles composed of mainly former ink (from E), with some Type I-circles at the top. (H) detail of the light gray Type I-circles in (E); apparently, the lighter outer ring may also have concentric structures, which are just not as distinct as when there was more ink between each subsequent ring. Photo credit: Dieter Buhl.

**Figure 3:**
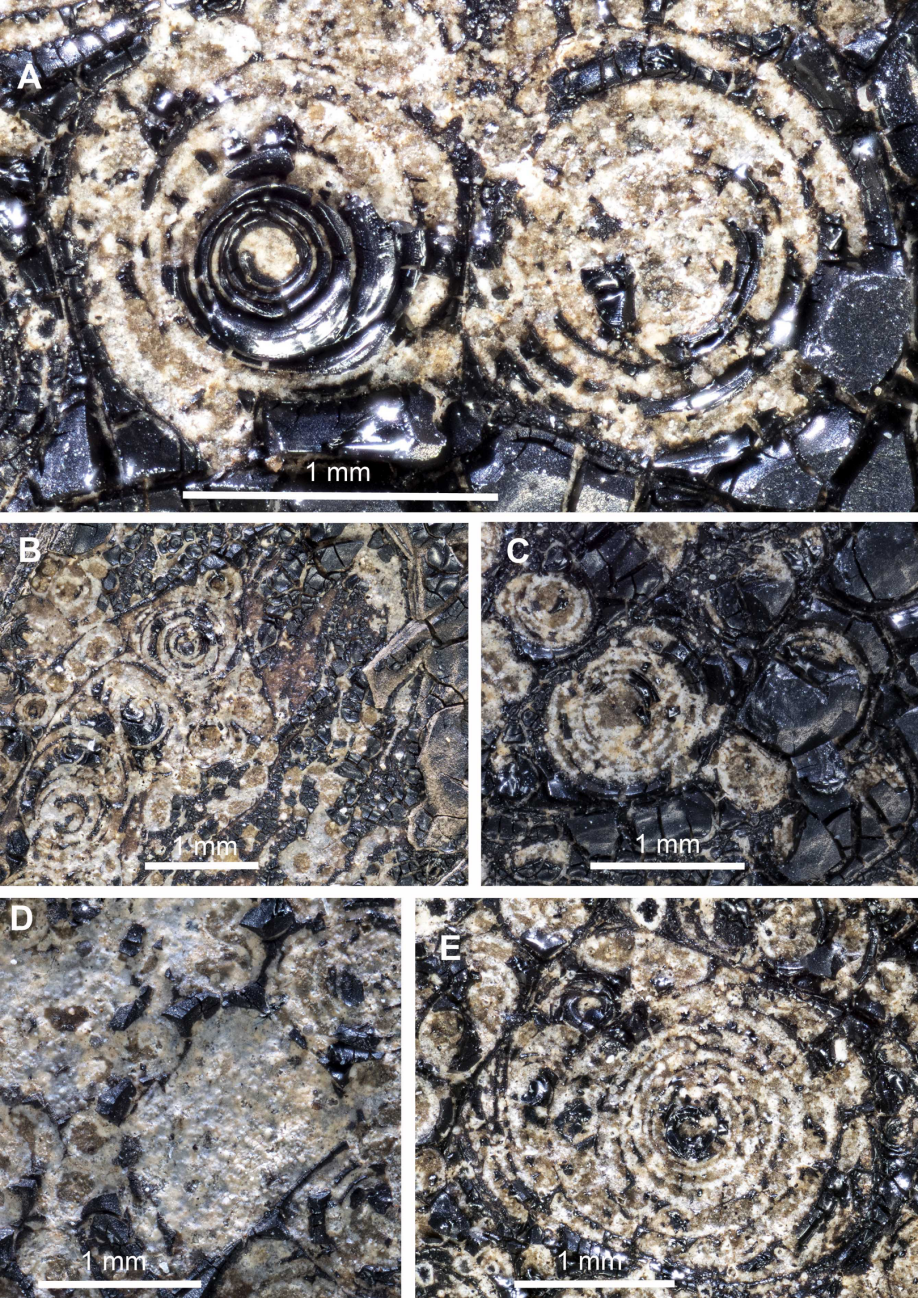
*Trachyteuthis hastiformis*, Kimmeridgian, Painten (Germany). SMNS 70496, leg. M. Kapitzke. Magnified details of Fig. 2. (A) Two Type II-circles; note how the ink forms a relief higher than the phosphate. (B) combination of Type I- and Type II-circles; note the arrangement in rows and the straight ink lines separating the rows. (C) Combination of Type I- and Type II-circles, overlain by a lot of ink. (D) Combination of Type I- and Type II-circles, some lacking the dark center or concentric circles. (E) Large Type II-circles, where the outer circles are filled with minute Type I-circles.

**Figure 4:**
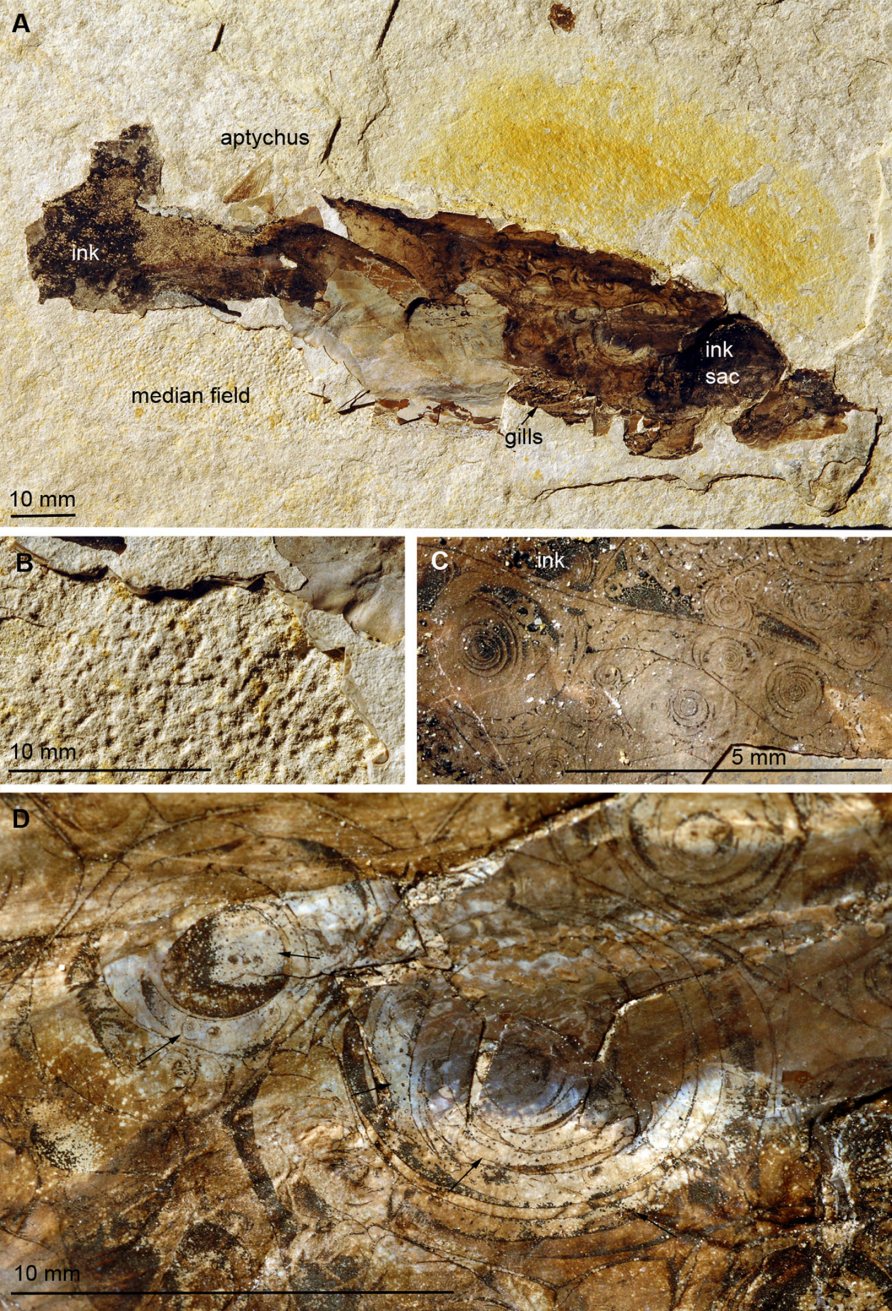
*Trachyteuthis hastiformis*, Kimmeridgian, Rygol quarry, Painten (Germany). PIMUZ 51761. (A) Overview. (B) Detail of the imprint of the median field. (C) Detail of the Type II-circles close to the anterior end of the mantle; note the gagate-like ink. (D) Detail of Type II-circles showing self-similarity (i.e., structures of similar organization but in different size classes; arrows); the smaller circles are of Type I.

Type I (Fig. 1A): This is the most common type. The circles of Type I have an outer field of a lighter gray and a central field in brownish darker gray, although the color contrast between the two is low (Figs. 2C, 2E, 2H and 3B). The central brownish circle appears to contain slightly more of some dark matter. The outer ring appears to be composed of more or less homogeneous phosphate, but sometimes, faint concentric circles are visible. This suggests that the concentric structures are omnipresent but only become visible in the presence of a black substance we interpret as ink: all examined coleoids had ink sacs, which are discernible in two of the specimens. The visual comparison of the black substance with ink in ink sac of other fossil coleoids supports the interpretation as ink (cf. Doguzhaeva, Mapes & Mutvei, 2002; Glass et al., 2012; Lindgren et al., 2012; Košťák et al., 2018).

Type II (Figs. 1B and 1C): The second form is richer in organic matter (former ink) and displays fine black concentric circles in the more deeply weathered gray phosphate (Figs. 2D, 2F, 3A and 3E). Usually, there is a darker spot in the center, surrounded by concentric or eccentric rings (i.e., the center of concentric rings either coincides with the outline of the circle or not), partial rings or spirals. In the largest Type II-circles, small Type I or even Type II-circles are present (Fig. 2F).

Type III (Fig. 1D): The third form appears to be composed of almost exclusively black organic matter (Figs. 2E and 2G), that is, former ink, but it shows similarly sized concentric circles as the Type II-circles. This type was found only in this specimen and only in a few spots. We assume that, although they appear nearly entirely black, they still contain quite some phosphate. This is supported by the presence of the slightly brighter rings in Fig. 2G.

These three types reflect an increase in organic matter, that is former ink and thus melanin. Especially around and posterior to the aptychi (=calcified lower jaw of an ammonoid that was eaten by the coleoid), spaces between circles are filled with black amorphous matter, which fractures like gagate (=fossil wood remains, bituminous coal, fossil hydrocarbon, also known as jet or black amber) but lacks internal circles.

### *Trachyteuthis* sp. from the Kimmeridgian of Painten (SMNS 70496; Figs. 2 and 3)

Like many specimens from Painten, it broke when the plate was split. The fossil is about 140 mm long. Like the other specimens, much of it is phosphatized. In the middle, a lanceolate field is covered by irregularly distributed black organic matter. This field might correspond to the gladius or its median field, although no ornament is discernible. Within this field, the circular structures occur (Fig. 2A). An elongate structure filled with organic matter turns upwards and to the left and might represent the ink duct. The posterior end of the supposed ink sac is accompanied by two elongate brownish fields, which show an oblique striation. Possibly, these are gill remains. Posterior to the ink duct, a lamellaptychus is embedded in the phosphate. Its surrounding suggests that this lower jaw of an ammonite was indeed inside the digestive tract of the coleoid (Fig. 2B). The circular structures are described in detail in the methods section since it is used as an example for the classification of circles.

### *Trachyteuthis* sp. from the Kimmeridgian of Painten (SMNS 70496, PIMUZ 51761; Fig. 4)

This is another fragmentary specimen of *Trachyteuthis*, which shows much of the gladius and non-mineralized remains. It is 133 mm long, preserves parts of the characteristic gladius (partially as imprint showing the granular ornament), phosphatized soft-tissues (mantle and gill remains), as well as the ink sac and ink. It is accompanied by ammonite aptychi (not as stomach contents). Like the other specimen, a lot of ink has leaked into the body of the coleoid during necrolysis or following an injury (no evidence available to determine, which process was involved).

This specimen shows the circles in a broad size range from less than 1 mm to about 10 mm (Figs. 4B–4D). The circles are visible, where the fossil remains were darkened by ink that seeped out of the ink sac. The circles are mostly concentric, often slightly eccentric with alternating broader grayish to brownish fields separated by finer dark brown to black lines. In the largest Type II-circles, the outer bands contain much smaller Type I-circles themselves (Fig. 4D, arrows). This self-similarity corroborates the self-organisation of these patterns. Additionally, the single circular fields vary in width both compared to their inner and outer neighboring rings and within a single field, which occasionally wedge out. The smaller-scale circles of less than 1 mm diameter tend to be of Type I while all larger ones belong to Type II.

### *Dorateuthis syriaca* from the Cenomanian of Haqel (PIMUZ 51762; Fig. 5)

This species represents the most common coleoid from Lebanon (Fuchs & Larson, 2011). The specimen described here (PIMUZ 51762) is 200 mm long and complete including most soft parts. Both slab and counterslab are available. Some arms, the eyes, mantle remains, ink sac and duct as well as the pen are well discernible. Like in the two *Trachyteuthis* described above, the circles are visible where the phosphatized mantle (characteristic transverse striations) remains show a brown staining from leaked ink. This area corresponds to a surface, where parts of the mantle musculature broke off when the slab was split.

**Figure 5:**
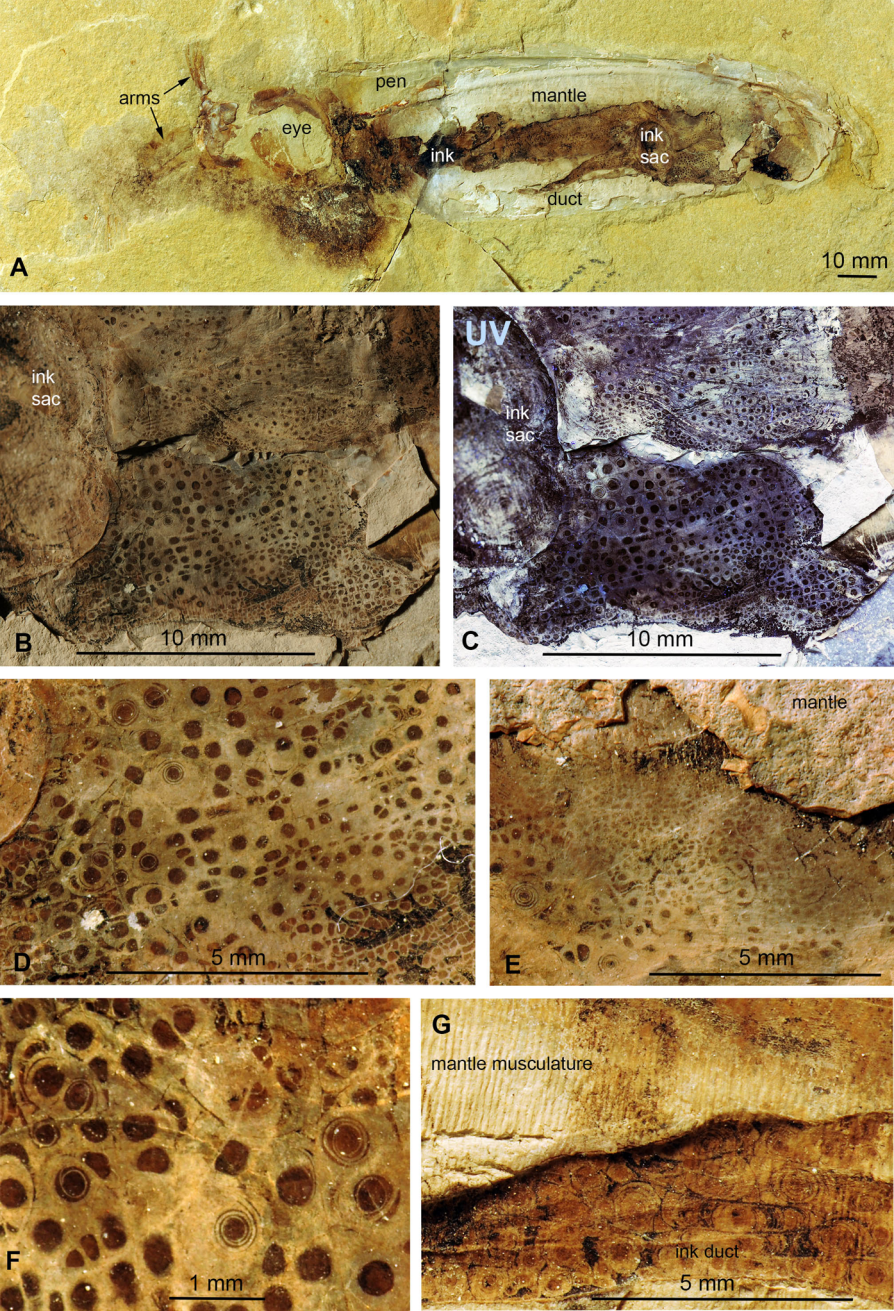
*Dorateuthis syriaca*, Cenomanian, Haqel (Lebanon). PIMUZ 51762. (A) Overview. (B and C) Detail behind ink sac under white (B) and UV-light (C). (D and F) Detail of B, mainly with Type I-circles posterior of ink sac; some are just dark dots, some Type II-circles with spirals, some with concentric circles; (F) Detail of D. (E) Change in Type I-circle size posterodorsal to the ink sac. (G) Mantle muscles (top) and ink duct with Liesegang ring-like Type II-circles (bottom).

Here, the circles are visible in and around the imprint of the ink sac as well as in and around the ink duct. This arrangement corroborates the genetic link between the circles and leaked ink. Here, the circles also occur in a certain range of sizes, although the largest ones are smaller than the largest ones in the Jurassic *Trachyteuthis*. In this *Dorateuthis*, most circles measure less than 1 mm and only a few circles reach about 5 mm (Figs. 5B and 5C). Posterior to the ink sac, most circles belong to Type I and are light gray with a darker brownish center. At the border between the two parts, finer lines occur forming either a couple of fine concentric circles or thin spiral lines completing a few turns (Fig. 5F), thus representing rather Type II-circles. Type III-circles were not seen in this specimen.

Behind the ink sac, the circles are grouped (Figs. 5B, 5C and 5E). Towards the margins of some of these fields, circle size gradually decreases as visible in Fig. 3E towards the top. Possibly, this reflects a link between a change in thickness of the tissue, in which the circles formed.

In the ink duct, Type II-circles are arranged in longitudinal fields, which are delimited by fine and distinct dark lines. In some of these fields, circles are surrounded by segments of surrounding circles covering up to about five mm in diameter (Fig. 5G). Similar longitudinal arrangements of the circles in fields delimited by bright lines are visible in Figs. 5B–5F. The surrounding overlying mantle (top third of the specimen in Fig. 5A) displays its transverse muscular striation but partially lacks the circles where it is whitish (no ink had penetrated this tissue). Further dorsally, the mantle is stained with ink, displaying more Type I-circles.

Under ultraviolet illumination, the dark parts of the circles stay dark, confirming the assumption that these parts are rich in organic carbon and contain less phosphate (Fig. 5C). For details of the composition, see the chapter “Chemical Analyses”.

### *Medusites capillaris* from the Kimmeridgian of Nusplingen (Fig. 6)

**Figure 6:**
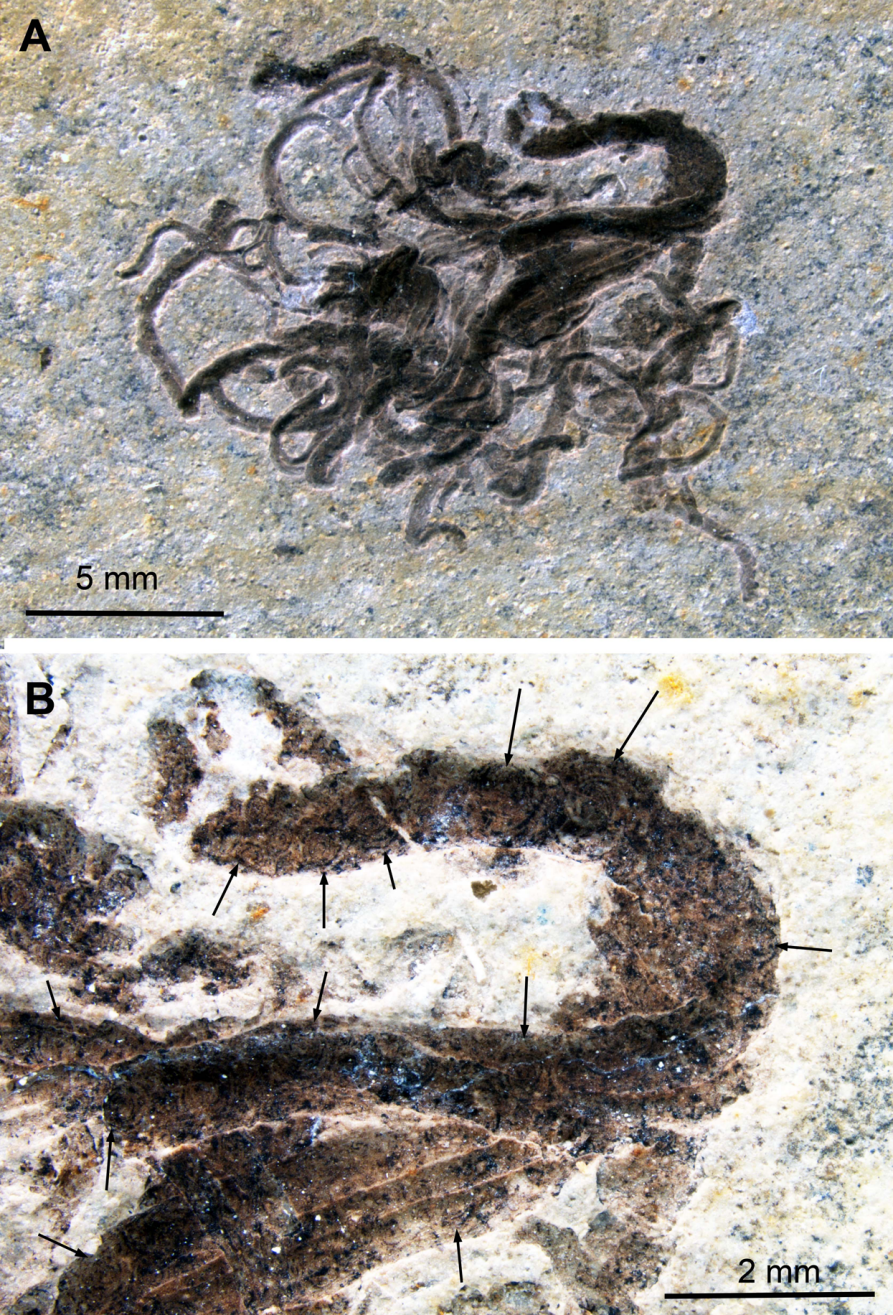
*Medusites capillaris*, Kimmeridgian, Nusplingen (Germany). SMNS P1856. (A) Overview. (B) Detail of coprolite with mostly Type II-circles (arrows); contrast strongly enhanced to make the circles visible.

This specimen differs from the others in not being a coleoid and the possible absence of ink. According to Schweigert (2001), *Medusites* is a fish coprolite. Nevertheless, its name reflects its earlier interpretation as a fossil jellyfish (Germar, 1827). The specimen depicted here measures 32 mm across and consists of an aggregate of elongate threads, which are between 0.2 and 2 mm thick. Especially the thickest parts display the Type II-circles, which were described in the coleoid fossils above. Here, the circles are much less well discernible and range between 0.1 and 1 mm in diameter. In this specimen, the circles are dark brown with an almost black center and fine black concentric lines in varying number (Fig. 6B). The number of concentric circles appears to depend on the size of the respective structure.

We did not carry out chemical analyses on this fossil. Since it is considered a fish coprolite, it is possible that the fish fed on coleoids. Consequently, the black matter might indeed be digested ink, but we cannot rule out that it is other organic matter or melanin of a different origin such as visual systems (Schäfer, 2013), liver (Scalia et al., 1990) or others (Wakamatsu & Ito, 2002), which complicates the recognition of original coleoid ink remains. The main mass of vertebrate coprolites is usually rich in phosphate and thus also probable for this specimen (corroborated by the color).

## Chemical Analyses

The question for the formation of the circular structures is linked with its composition. Visual clues such as color and breakage as well as occurrence in distinct organs pointed at calcium phosphates in the case of light gray and light brownish substances. Correspondingly, the spatial proximity of darker parts to the ink sac and ink duct imply that it is former ink staining these areas. Localities with Mesozoic conservation deposits such as Nusplingen and Painten in Germany as well as Sahel Alma, Hajula and Haqel in Lebanon are known to have the potential of preserving eumelanin (for analyses of eumelanin from the United Kingdom see Glass et al., 2012).

**Figure 7:**
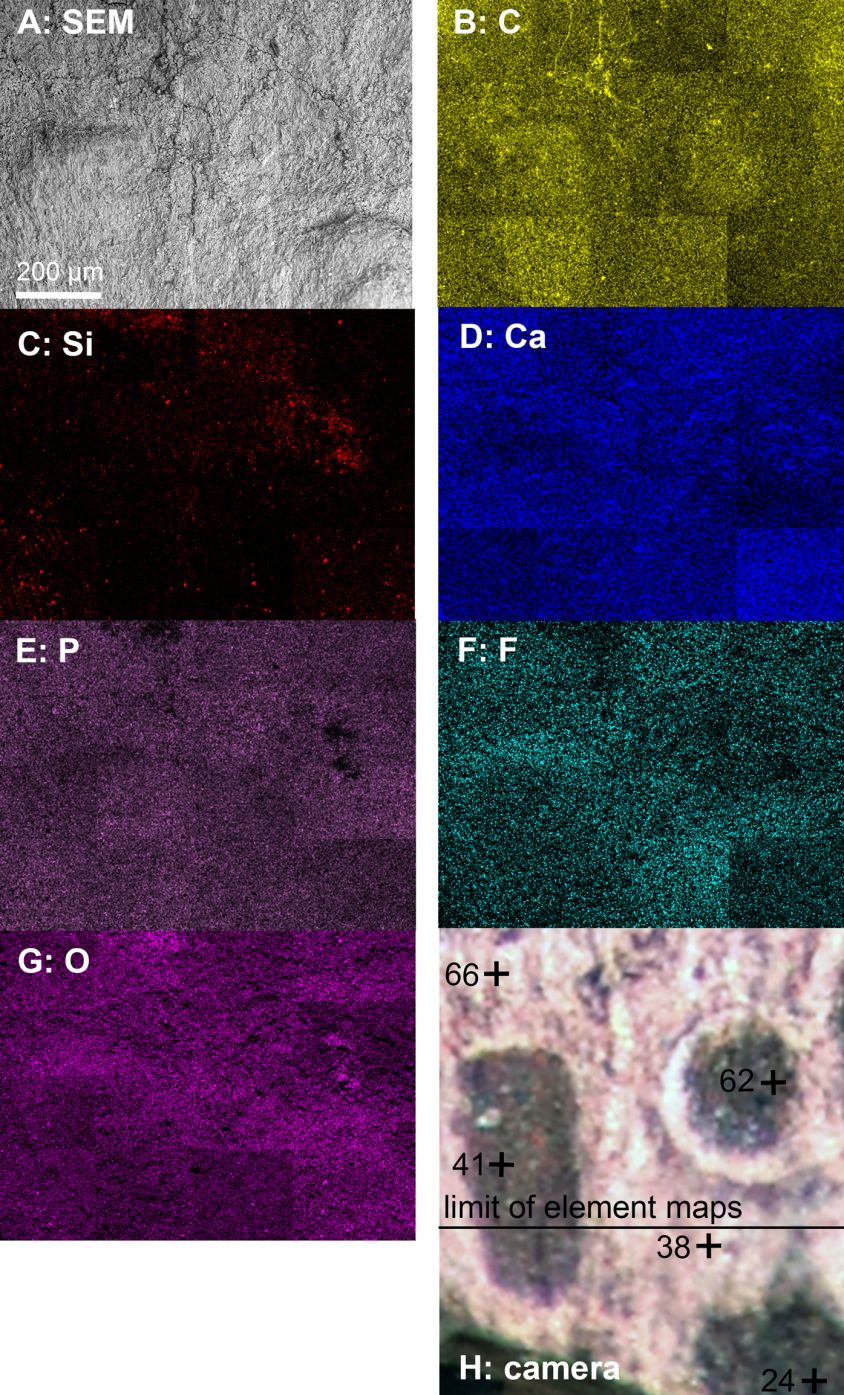
EDX-element maps of some Type I-circles of *Dorateuthis hastiformis*, Cenomanian, Haqel (Lebanon). PIMUZ 51762. Image width ca. 1 mm. (A) SEM-image. (B–G) Maps of the indicated elements. (H) Photo made under visible white light.

**Figure 8:**
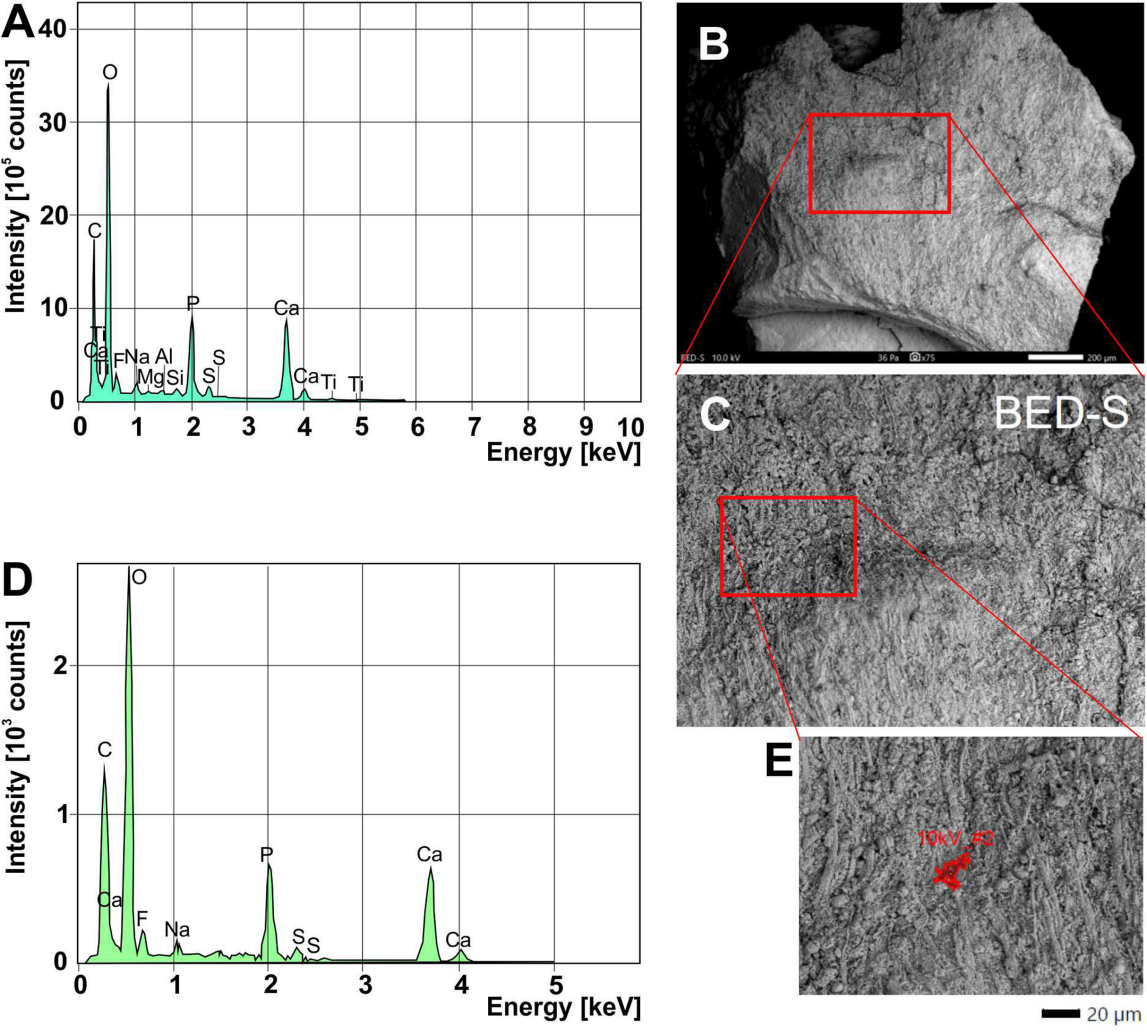
EDX-element analyses on a chip taken from *Dorateuthis syriaca*, PIMUZ 51762, from the Cenomanian of Haqel (Lebanon). (A) EDX-spectrum measured on the whole surface are shown in C (red rectangle). (B) Overview of the sample taken with SEM (white light photo in Fig. 7H; the red rectangle/box corresponds partly with that part); scale bar is 200 microns. (C) Close-up of the area marked with the red box in B. (D) EDX-spectrum measured on the surface area shown in E. (E) Detail of C, showing where the spectrum in D was measured. The elongate fibers are likely muscle fibers from the mantle musculature. The small spheres maybe be phosphatized bacteria; for melanosomes, they are too large.

**Figure 9:**
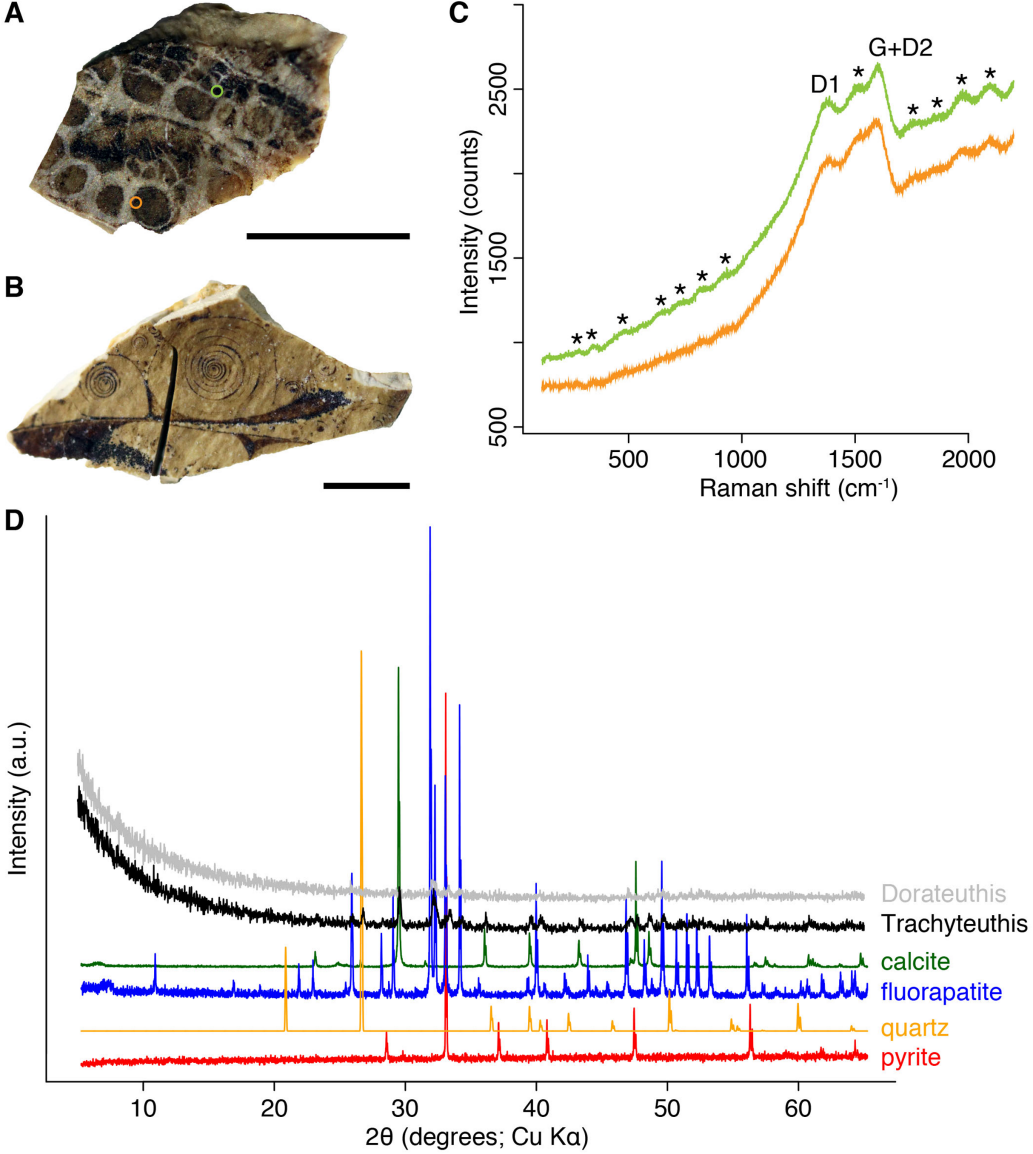
Composition of the Liesegang rings resembling chromatophores in two Mesozoic coleoids. (A) Millimetric sample of *Dorateuthis syriaca*, Cenomanian, Haqel (Lebanon). PIMUZ 51762. (B) Millimetric sample of *Trachyteuthis hastiformis*, Kimmeridgian, Rygol quarry, Painten (Germany). PIMUZ 51761. (C) Raman spectroscopy (using a 532-nm laser) of the circles in the *Doratheutis* sample, showing the so-called graphite (G) and defect (D) bands typical of organic materials preserved in sedimentary rocks. The bands identified by an asterisk are instrumental quasi-periodic ripples resulting from intense background luminescence (see Alleon et al. (2020) and references therein). (D) Diffractograms obtained for both samples, compared to references for minerals commonly associated with fossils. Phase identification confirms the phosphatic nature of these structures; only apatite is found in the *Doratheutis* sample, quartz and calcite from the sedimentary matrix are found in association with apatite in the *Trachyteuthis* sample. Scale bars, 1 mm.

We analyzed a sample from the Lebanese *Dorateuthis* (PIMUZ 51762; Fig. 5). Two of the circles were examined by EDX and Raman spectroscopy using various wave-lengths. The latter did provide only little results due to strong fluorescence, which is typical of, for example, fluorapatite. Elemental mapping by EDX was carried out on the original sample surface in two of the circles, and revealed a slight increase in carbon in the dark parts of Type I-circles. Figure 7 shows the EDX elemental maps with the somewhat higher C-content in the brown centers, while O and possibly F appears to be slightly elevated in the lighter gray parts. Two EDX-spectra of the same sample are shown in Fig. 8. As far as the weight percentages are concerned, the differences in Ca and C are confirmed by the values in Table 1. These analyses revealed that calcium phosphate is predominant in the light gray circles behind the ink sac. In the dark center, C appears to be slightly enriched (Fig. 7), while Ca is a bit more abundant in the light gray parts (Table 1). Raman spectroscopy confirms, despite a strong fluorescence associated to a quasi-periodic ripple-artefact (Alleon et al. (2020) and references therein), the presence of C in the circles evidenced by the so-called graphite (G) and defect (D) bands typical of fossil organic materials (Fig. 9C). However, no carbon signal was obtained in the light gray parts at the margin of the Type I-circles. By contrast, X-ray diffraction carried out over a millimetric sample extracted from the fossil confirms the phosphatic nature (apatite) of these structures (Fig. 9D). Quartz and calcite from the sedimentary matrix are found in association with apatite in the German *Trachyteuthis* (Fig. 9D).

**Table 1:** Results of EDX-analyses in some Type I-circles in *Dorateuthis syriaca*, Cenomanian, Haqel (Lebanon), PIMUZ 51762. Data gathered by A. Kaech. Values in volume percent. Values in the gray columns were obtained from the dark brownish surfaces, those in the white columns from the light gray surfaces. Position of samples (numbers in black with a +): See Fig. 7H.

Sample nr.	24	38	41	62	66	Ø brown	Ø gray
O	46.16	37.60	40.82	41.71	47.10	42.90	42.35
C	22.17	11.16	13.97	18.73	14.70	**18.29**	**12.93**
Ca	21.98	34.14	28.39	24.61	28.31	**24.99**	**31.23**
P	5.73	11.84	10.42	8.7	5.21	8.28	8.53
F	2.11	3.03	3.74	2.99	1.86	2.95	2.45
S	1.03	1.42	1.43	1.54	0.71	1.33	1.07
Na	0.50	0.69	0.93	0.70	0.52	0.71	0.61
Si				0.81	1.12		

**Note:**

The values in bold highlight the average values of C and Ca, which differ between the dark and the gray parts of the circles.

## Discussion

It is interesting to note that none of the previous authors dealing with phosphatized soft-tissue preservation of cephalopods or other marine animals, preserved ink sacs, or the process of phosphatization mentioned these peculiar circles and spiral structures (Allison, 1988; Wilby et al., 2004; Fuchs, 2006a, 2006b; Fuchs & Larson, 2011; Dornbos, 2011; Donovan, Fuchs & Part, 2016; Klug et al., 2015, 2016). In order to understand their formation, it is appropriate to compare the structures with at least superficially similar structures such as silicification rings as well as Liesegang rings.

### Silicification rings

**Figure 10:**
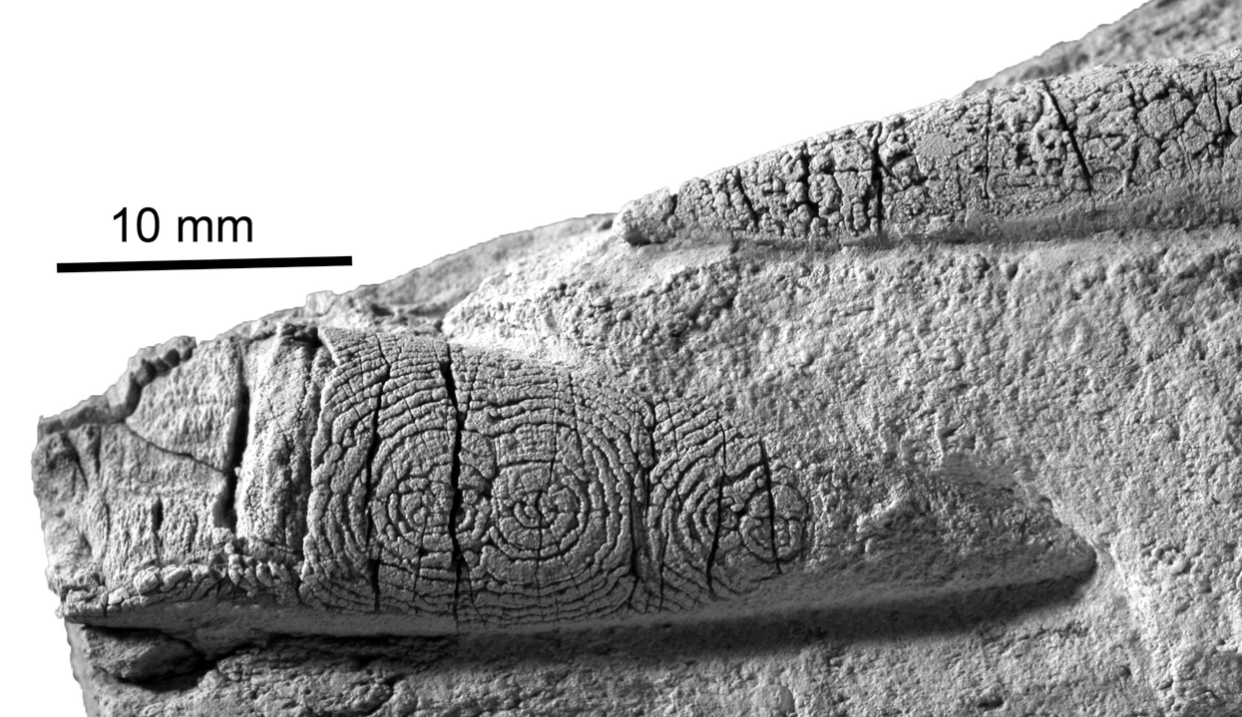
The belemnite *Neohibolithes* from the Altmann–Member, Barremian, southwestern Altmann–Sattel, Alpstein Massif (Switzerland). Specimen NMSG Coll. P. Kürsteiner 5A.05.07. Silicification rings on the surface of the rostrum. Specimen whitened with NH_4_Cl. Note the thin silica crust with underlying original calcite (termed by some “shelter preservation”). Also, the centers are slightly fuzed which was never observed in our material.

The concentric structures described here from fossil tissues of Mesozoic coleoids and one coprolite superficially resemble silicification rings or beekite rings as widely known from fossils with originally calcitic shell material (Fig. 10) and were first described by Von Buch (1828). Our chemical analyses as well as the optical appearance suggest that no or very low amounts of silica (around 1 weight percent; Table 1) are present in the fossils under consideration here.

Nevertheless, as we will discuss below, the concentric and circular shapes seen in our specimens are highly reminiscent of beekite rings (Butts & Briggs, 2011; Butts, 2014). For these structures, it has been suggested that episodically changing silica availability or varying calcite dissolution account for the formation of Liesegang-like concentric siliceous rings (Hodges, 1932; Holdaway & Clayton, 1982). This similarity hints at genetic parallels, that is, reaction-diffusion processes (Dietrich, 2019), which are likely linked with a gel-like state during early diagenesis.

### Liesegang rings

**Figure 11:**
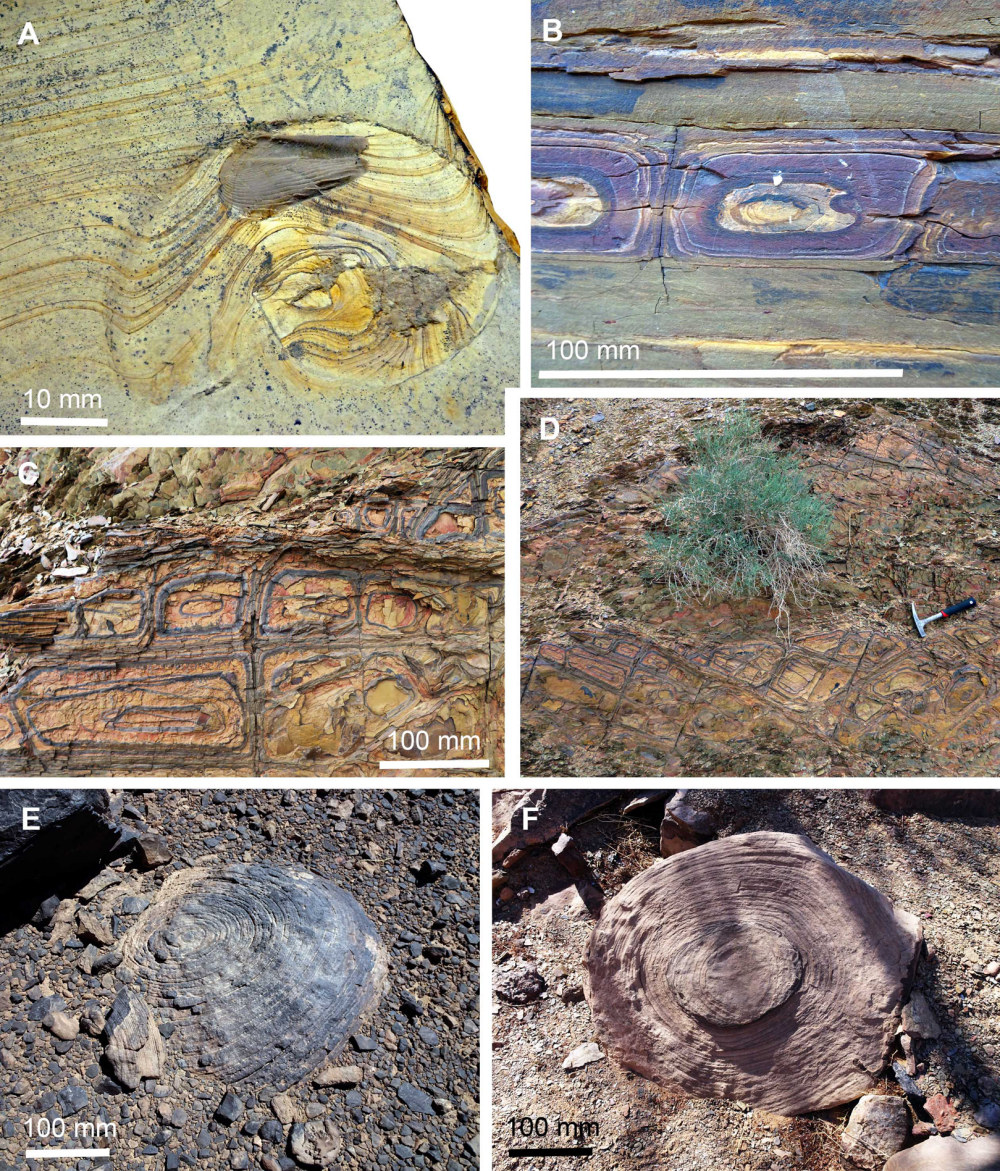
Liesegang rings in sediments and fossils. (A) *Neochetoceras* cf. *mohri* with lamellaptychus in the body chamber, Tithonian, Late Jurassic, Eichstätt, Germany; Liesegang rings deflected by the topography created by the collapsed ammonoid conch and the aptychus. (B–D) Liesegang rings in Fe-rich sediments of the Famennian near Madene El Mrakib, Morocco. (B) Section through bedding. (C and D) bedding plane; (C) detail of D; D, hammer (300 m) for scale. (E and F) Large sandstone boulders, probably correlate of the Hangenberg Sandstone, latest Famennian, near Madene El Mrakib, Morocco; note the desert varnish in E and the differential weathering depending on mineral content in F.

Liesegang rings are concentric structures that form by chemical precipitation, often depending on the concentration of solutions of poorly soluble salts and commonly occur in gel-like environments (Hodges, 1932; Keller & Rubinow, 1981; Zhang et al., 2014). There is quite a considerable number of papers discussing the processes in the experimental formation of Liesegang rings with various salts including carbonates, chromates, metal hydroxides, sulfides but also sulfates (Swami & Kant, 1966; Zhang et al., 2014). The formation of Liesegang rings is, however, also widely known from biological systems (Islam Ou et al., 2012) and geological systems (Seilacher, 2001) as shown in Fig. 11.

Some phenomenona have been observed repeatedly in different experimental systems. For example, the ring width tends to depend on the distance to the gel-liquid interface and the rings apparently do not move. The findings by Zhang et al. (2014) are of special relevance for the system discussed here, because they obtained CaHPO_4_ by applying CaCl_2_ and Na_2_HPO_4_ to a gelatin ball, that is, there is some chemical similarity. Their main discoveries were the important role of the inner electrolyte Ca^2+^, which increased gelatin viscosity and allowed the formation of Liesegang rings. Remarkably, Ca is more abundant outside the circles found in the fossil coleoids. Zhang et al. (2014: p. 604*)* also found that “*the interaction of Ca*^*2+*^ *and –COO*^*−*^ *of gelatin chains […] dominated the formation of CaHPO*_*4*_ *LRs in gelatin*” and “*the time law, spacing law and width law observed in 1D/2D gel systems were obeyed in this 3D gelatin system. The interaction of Ca*^*2+*^ *and HPO*_*4*_^*2−*^ *with gelatin matrix played a key role to the formation of LRs due to the existence of carboxylic groups on the gelatin chains*”.

How can this knowledge be transferred to the fossils described here? We cannot reconstruct all chemical details of the diagenetic processes that occurred during the formation of Liesegang rings of these fossil coleoids. In the following, we propose a diagenetic pathway that generated these pseudo-chromatophores.

In the course of necrolysis, soft parts begin to fall apart (disarticulate). In the decay experiments of Clements et al. (2016), the mantle tissue started to fracture into blocks quite early. This process began already after a few days, while large pieces of mantle remained intact for weeks. After 7–9 days, the ink sac disintegrated. This is relevant because it informs us about the earliest onset of the formation of Liesegang rings. Only then, ink seeped into the surrounding tissues (for chemical analyses of fossil coleoid ink see Glass et al. (2012)). Remarkably, it was after slightly more than 6 days that the pH in the decaying octopus reached the phosphatization window (Clements et al., 2016: p. 7; Briggs & Wilby, 1996). This suggests that, under normal conditions where there is no environmental factor to retard decay (i.e., low temperature—Briggs & Kear (1993) for variables that effect decay rates), Liesegang rings must have started to form very early during decay—possibly a week after the coleoids’ death and burial.

For the Liesegang rings to form, a gelatinous matrix is needed. Muscle mass loses ultrastructure within days (Kear, Briggs & Donovan, 1995) and becomes “gellified” rapidly during decay (T. Clements, 2016, personal observation). Calcium phosphate was available in the sea water in supersaturation. However, Ca^2+^ and HPO_4_^2−^-ions were also available in the soft-tissues of the coleoids (Clements et al., 2016). Likely, there was a favorable gradient in these ions, allowing the Liesegang rings to form. The rhythmic secretion allowed then ink to settle according to the gradients within the Liesegang rings and ink availability as well as space between the tissue parts that were being phosphatized.

## Conclusion

We describe two Jurassic and one Cretaceous octobrachian coleoid as well as a Jurassic coprolite, which display subcircular structures with concentric ring patterns. Superficially, they resemble chromatophores, but their fine structure and proportions falsify this interpretation. According to their internal patterns, we differentiate between three types of circles. Type I displays a light gray circle with a brownish center, in Type II, dark, more or less concentric rings and spirals surround the center, while in Type III, the abundance of ink colored the circles more or less completely in black.

These patterns suggest that they are Liesegang rings, which are diagenetic structures well-known from chemical experiments with a broad range of molecules, from biological systems and geological structures. Published results of experiments with calcium phosphates in gelatin revealed that phosphates can form such rings as well. The fossils documented here were all rich in calcium and phosphates as well as organic matter. During decay, we speculate that the matrix, where the Liesegang rings formed, reached a gel-like state. Apparently, the concentrations of dissolved Ca^2+^ and HPO_4_^2−^-ions were such that Liesegang rings formed. Depending on the space between adjacent mantle blocks that became phosphatized, squid ink (or other organic matter in the case of the coprolite) could enter and visually enhanced the Liesegang rings to varying degrees, causing the formation of the three types of circles. This study shows how some putative biological structures seen in fossils have formed by taphonomic processes and thus great care has to be taken in the interpretation of unknown fossil structures.

## Additional Information and Declarations

### Data Availability

The following information was supplied regarding data availability: All measurements and data are included in the article. Specimens are stored in the following museums: NMSG-Naturmuseum Sankt Gallen, Switzerland; PIMUZ-Paläontologisches Institut und Museum, Universität Zürich, Switzerland; SMNS-Staatliches Museum für Naturkunde, Stuttgart, Germany. Specimen accession numbers are available in the Material section: PIMUZ 51761; SMNS 70496; SMNS P1856.
